# Challenges and Controversies in the Genetic Diagnosis of Hereditary Spastic Paraplegia

**DOI:** 10.1007/s11910-021-01099-x

**Published:** 2021-02-28

**Authors:** Lydia Saputra, Kishore Raj Kumar

**Affiliations:** 1 Northern Beaches Hospital, Frenchs Forest, New South Wales Australia; 2 Garvan Institute of Medical Research, Darlinghurst, New South Wales Australia; 3 Molecular Medicine Laboratory, Concord Repatriation General Hospital, Concord, Sydney, New South Wales Australia; 4 Sydney Medical School, University of Sydney, Sydney, New South Wales Australia; 5 Institute of Precision Medicine & Bioinformatics, Sydney Local Health District, Royal Prince Alfred Hospital, Camperdown, New South Wales Australia

**Keywords:** Hereditary spastic paraplegia, HSP, Diagnosis, Genetics, Whole-exome sequencing, Whole-genome sequencing

## Abstract

**Purpose of Review:**

The hereditary spastic paraplegias (HSPs) are a group of disorders characterised by progressive lower limb weakness and spasticity. We address the challenges and controversies involved in the genetic diagnosis of HSP.

**Recent Findings:**

There is a large and rapidly expanding list of genes implicated in HSP, making it difficult to keep gene testing panels updated. There is also a high degree of phenotypic overlap between HSP and other disorders, leading to problems in choosing the right panel to analyse. We discuss genetic testing strategies for overcoming these diagnostic hurdles, including the use of targeted sequencing gene panels, whole-exome sequencing and whole-genome sequencing. Personalised treatments for HSP are on the horizon, and a genetic diagnosis may hold the key to access these treatments.

**Summary:**

Developing strategies to overcome the challenges and controversies in HSP may hold the key to a rapid and accurate genetic diagnosis.

## Introduction

The hereditary spastic paraplegias (HSPs) are a group of conditions characterised by progressive weakness and spasticity of the lower limbs [1, 2]. They can have autosomal dominant (AD), autosomal recessive (AR), X-linked and mitochondrial modes of inheritance [3]. The HSPs can be classified as either ‘pure’ (uncomplicated) or ‘complex’ (complicated). Pure forms involve lower limb spastic paraplegia and may include bladder involvement and subtle sensory signs such as impaired vibration sense. Complicated forms include additional neurological and non-neurological manifestations, such as cognitive impairment, dysarthria, optic atrophy and peripheral neuropathy [1]. There are also syndromic forms such as Silver syndrome (spastic paraparesis with distal amyotrophy predominantly of the hands). The different genetic forms are assigned spastic paraplegia loci (SPG), although the HSP genes may also be listed according to the new MDSGene nomenclature, e.g. SPAST-HSP for SPG4 [4]. The prevalence of AD HSP ranges from 0.5 to 5.5 per 100,000 and that of AR HSP from 0.3 to 5.3 per 100,000 [5]. Although the HSPs are rare, the progressive and disabling nature of these disorders means that they warrant greater attention from clinicians and researchers.

In this review, we discuss current challenges to reach a genetic diagnosis in HSP. These include (i) the large number of genes involved and the rapid rate of gene discovery, (ii) major phenotypic overlap between HSP and other disorders and (iii) disorders that mimic HSP. Further adding to the complexity is that a single HSP gene can have different patterns of inheritance, for example both autosomal dominant and recessive. Additionally, a single patient with HSP can have concurrent independent genetic diagnoses. Moreover, pseudodominant inheritance of autosomal recessive disease can occur when an individual with mutations on both copies of the gene has a partner carrying a heterozygous mutation, which may result in an affected offspring, a situation that typically occurs when there is a high carrier frequency in the population. In light of these challenges, we discuss the pros and cons of common genetic testing strategies in HSP such as multi-gene panels, whole-exome sequencing (WES) and whole-genome sequencing (WGS). An accurate, timely genetic diagnosis in HSP may become particularly relevant as new, targeted therapies are on the horizon.

## Challenges to a Genetic Diagnosis

### Multiple Genes and a Rapidly Increasing Gene List

There are many genes causative of HSP resulting in a high level of genetic heterogeneity. Different forms of HSP are assigned a genetic locus according to the order in which they are discovered (spastic paraplegia loci, SPG). Currently, the Online Mendelian Inheritance in Man (OMIM) lists 81 distinct genetic forms of HSP (Table 1, excluding SPG40 and for SPG65 see SPG45). Of these 81 genetic forms, 13 do not have a specific gene identified. Furthermore, while 55 had been identified in more than 1 family, twenty-six were reported in single families, warranting further confirmation.

**Table 1:** Summary of genetic forms of hereditary spastic paraplegia

Type	Gene	Location	Phenotype MIM number	Inheritance	Identified in more than 1 family with HSP (yes or no)	Allelic disorders/alternative gene-phenotype relationships, MIM number
SPG1	*L1CAM*	Xq28	MASA syndrome, CRASH syndrome, MIM303350	XLR	Yes	Partial agenesis of the corpus callosum, MIM308840; hydrocephalus due to aqueductal stenosis, hydrocephalus with congenital idiopathic intestinal pseudoobstruction, hydrocephalus with Hirschsprung disease, MIM307000
SPG2	*PLP1*	Xq22.2	MIM312920	XLR	Yes	Pelizaeus-Merzbacher disease, MIM312080
SPG3A	*ATL1*	14q22.1	MIM182600	AD	Yes	Hereditary sensory neuropathy type ID, MIM613708
SPG4	*SPAST*	2p22.3	MIM182601	AD	Yes	
SPG5A	*CYP7B1*	8q12.3	MIM270800	AR	Yes	Congenital bile acid synthesis defect type 3, MIM613812
SPG6	*NIPA1*	15q11.2	MIM600363	AD	Yes	
SPG7	*SPG7*	16q24.3	MIM607259	AR	Yes	
SPG8	*KIAA0196* (*WSHC5*)	8q24.3	MIM603563	AD	Yes	Ritscher-Schinzel syndrome 1, MIM220210
SPG9A, B	*ALDH18A1*	10q24.1	MIM601162, MIM616586	AD	Yes	AD cutis laxa 3, MIM616603; AR cutis laxa type IIIA, MIM219150
SPG10	*KIF5A*	12q13.3	MIM604187	AD	Yes	Neonatal intractable myoclonus, MIM617235
SPG11	*SPG11*	15q21.1	MIM604360	AR	Yes	Juvenile amyotrophic lateral sclerosis 5, MIM602099; axonal Charcot-Marie-Tooth disease type 2X, MIM616668
SPG12	*RTN2*	19q13.32	MIM604805	AD	Yes	
SPG13	*HSPD1*	2q33.1	MIM605280	AD	Yes	Hypomyelinating leukodystrophy 4, MIM612233
SPG14	*-*	3q27-q28	MIM605229	AR	No	
SPG15	*ZFYVE26*	14q24.1	MIM270700	AR	Yes	
SPG16	*-*	Xq11.2	MIM300266	XLR	No	
SPG17	*BSCL2*	11q12.3	Silver spastic paraplegia syndrome, MIM270685	AD	Yes	Progressive encephalopathy with or without lipodystrophy, MIM615924; congenital generalised lipodystrophy type 2, MIM269700; distal hereditary motor neuropathy type VA, MIM600794
SPG18	*ERLIN2*	8p11.23	MIM611225	AR	Yes	
SPG19	*-*	9q	MIM607152	AD	No	
SPG20	*SPG20*	13q13.3	Troyer syndrome, MIM2759002	AR	Yes	
SPG21	*SPG21*	15q22.31	MAST syndrome, MIM248900	AR	Yes	
SPG22	*SLC16A2*	Xq13.2	Allan-Herndon-Dudley syndrome, MIM300523	XL	Yes	
SPG23	*DSTYK*	1q32.1	MIM270750	AR	Yes	Congenital anomalies of kidney and urinary tract 1, MIM610805
SPG24	*-*	13q14	MIM607584	AR	No	
SPG25	*-*	6q23-q24.1	MIM608220	AR	No	
SPG26	*B4GALNT1*	12p11.1-q14	MIM609195	AR	Yes	
SPG27	*-*	10q22.1-q24.1	MIM609041	AR	No	
SPG28	*DDHD1*	14q22.1	MIM603940	AR	Yes	
SPG29	*-*	1p31.1-p21.1	MIM609727	AD	No	
SPG30	*KIF1A*	2q37.3	MIM610357	AD, AR	Yes	AD mental retardation type 9, MIM 614255; hereditary sensory neuropathy type IIC, MIM614213
SPG31	*REEP1*	2p11.2	MIM610250	AD	Yes	Distal hereditary motor neuronopathy type VB, MIM614751
SPG32	*-*	14q12-q21	MIM611252	AR	No	
SPG33	*ZFYVE27*	10q24.2	MIM610244	AD	No	
SPG34	*-*	Xq24-25	MIM300750	XLR	No	
SPG35	*FA2H*	16q23.1	MIM612319	AR	Yes	
SPG36	*-*	12q23-q24	MIM613096	AD	No	
SPG37	*-*	8p21.1-q13.3	MIM611945	AD	No	
SPG38	*-*	4p16-p15	MIM612335	AD	No	
SPG39	*PNPLA6*	19p13.2	MIM612020	AR	Yes	Laurence-Moon syndrome, MIM245800; Boucher-Neuhauser syndrome MIM215470; Oliver-McFarlane syndrome MIM275400
SPG40	-	-	-	AD	No	
SPG41	*-*	11p14.1-p11.2	MIM613364	AD	No	
SPG42	*SLC33A1*	3q25.31	MIM612539	AD	No	Congenital cataracts, hearing loss, and neurodegeneration, MIM614482
SPG43	*C19orf12*	19p13.11-q12	MIM615043	AR	No	Neurodegeneration with brain iron accumulation 4, MIM614298
SPG44	*GJC2*	1q42.13	MIM613206	AR	No	Hypomyelinating leukodystrophy 2, MIM608804
SPG45	*NT5C2*	10q24.3–q25.1	MIM 613162	AR	Yes	
SPG46	*GBA2*	9p13.3	MIM614409	AR	Yes	
SPG47	*AP4B1*	1p13.2	MIM614066	AR	Yes	
SPG48	*KIAA0415*	7p22.1	MIM613647	AR	Yes	
SPG49	*TECPR2*	14q32.31	MIM615031	AR	Yes	
SPG50	*AP4M1*	7q22.1	MIM612936	AR	Yes	
SPG51	*AP4E1*	15q21.2	MIM613744	AR	Yes	Familial persistent stuttering 1, MIM184450
SPG52	*AP4S1*	14q12	MIM614067	AR	Yes	
SPG53	*VPS37A*	8p22	MIM614898	AR	Yes	
SPG54	*DDHD2*	8p11.23	MIM615033	AR	Yes	
SPG55	*C12orf65*	12q24.31	MIM615035	AR	Yes	Combined oxidative phosphorylation deficiency 7, MIM613559
SPG56	*CYP2U1*	4q25	MIM615030	AR	Yes	Pseudoxanthoma elasticum
SPG57	*TFG*	3q12.2	MIM604484	AR	Yes	Hereditary motor and sensory neuropathy, Okinawa type, MIM604484
SPG58	*KIF1C*	17p13.2	AR spastic ataxia 2, MIM611302	AR	Yes	
SPG59	*USP8*	15q21.2	-	AR	No	
SPG60	*WDR48*	3p22.2	-	AR	No	
SPG61	*ARL6IP1*	16p12.3	MIM615685	AR	Yes	
SPG62	*ERLIN1*	10q24.31	MIM615681	AR	Yes	
SPG63	*AMPD2*	1p13.3	MIM615686	AR	No	Pontocerebellar hypoplasia type 9, MIM615809
SPG64	*ENTPD1*	10q24.1	MIM615683	AR	Yes	
SPG66	*ARSI*	5q32	-	AR	No	
SPG67	*PGAP1*	2q33.1	-	AR	No	AR mental retardation 42, MIM615802
SPG68	*KLC2*	11q13.1	Spastic paraplegia, optic atrophy, and neuropathy, MIM609541	AR	Yes	
SPG69	*RAB3GAP2*	1q41	-	AR	No	Martsolf syndrome, MIM212720; Warburg micro syndrome 2, MIM614225
SPG70	*MARS1*	12q13.3	-	AR	No	AD Charcot-Marie-Tooth disease type 2, MIM616280; interstitial lung and liver disease, MIM615486
SPG71	*ZFR*	5p13.3	-	AR	No	
SPG72	*REEP2*	5q31	MIM615625	AD, AR	Yes	
SPG73	*CPT1C*	19q13.33	MIM616282	AD	Yes	
SPG74	*IBA57*	1q42.13	MIM616451	AR	No	Multiple mitochondrial dysfunctions syndrome 3, MIM615330
SPG75	*MAG*	19q13.12	MIM616680	AR	Yes	
SPG76	*CAPN1*	11q13.1	MIM616907	AR	Yes	
SPG77	*FARS2*	6p25.1	MIM617046	AR	Yes	Combined oxidative phosphorylation deficiency 14, MIM614946
SPG78	*ATP13A2*	1p36.13	MIM617225	AR	Yes	Kufor-Rakeb syndrome, MIM606693
SPG79	*UCHL1*	4p13	MIM615491	AR	Yes	?Parkinson disease 5, susceptibility to, MIM613643
SPG80	*UBAP1*	9p13.3	MIM618418	AD	Yes	
SPG81	*SELENOI*	2p23.3	MIM618768	AR	Yes	
SPG82	*PCYT2*	17q25.3	MIM618770	AR	Yes	
SPG83	*HPDL*	1p34.1	MIM619027	AR	Yes	
Unassigned	*RNF170*	8p11.21		AR	Yes	AD sensory ataxia 1, MIM608984
Unassigned	*FAR1*	11p15.3		AR	Yes	Peroxisomal fatty acyl-CoA reductase 1 disorder, MIM616154
Unassigned	*NEFL*	8p21.2		AR	Yes	Charcot-Marie-Tooth disease, dominant intermediate G, MIM 617882; Charcot-Marie-Tooth disease type 1F, MIM607734; Charcot-Marie-Tooth disease type 2E, MIM607684
Unassigned	*VPS13D*	1p36.22-p36.21		AR	Yes	Spinocerebellar ataxia, autosomal recessive 4, MIM607317
Unassigned	*TUBB4A*	19p13.3		AD	Yes	Dystonia 4, MIM128101; hypomyelinating leukodystrophy 6, MIM612438
Unassigned	*VCP*	9p13.3		AD	Yes	Charcot-Marie-Tooth disease type 2Y, MIM 616687; frontotemporal dementia and/or amyotrophic lateral sclerosis 6, MIM613954; inclusion body myopathy with early-onset Paget disease and frontotemporal dementia 1, MIM167320
Unassigned	*POLR3A*	10q22.3		AR	Yes	Hypomyelinating leukodystrophy 7 with or without oligodontia and-or hypogonadotropic hypogonadism, MIM607694; Wiedemann-Rautenstrauch syndrome, MIM264090

Information extracted from OMIM [6]

*AD*, autosomal dominant; *AR*, autosomal recessive; *XLR*, X-linked recessive

Due to the rapid rate of progress of HSP research, new genes are being identified on a regular basis. Examples of recently identified HSP genes include *UCHL1* (SPG79), *UBAP1* (SPG80), *SELENOI* (SPG81), *PCYT2* (SPG82), *HPDL* (SPG83), and those not yet assigned a locus (*RNF170* and *FAR1*) [7–16]. Some genes are much rarer than others, and it cannot be excluded that certain mutations may be ‘private’ to individual families. For example, a *SCL33A1* mutation was implicated as a cause of AD HSP (SPG42) in a large Chinese pedigree [17], but mutations in this gene were not identified in a large sample of European HSP cases [18]. In contrast, multiple groups have reported that *UBAP1* causes AD HSP with a pure phenotype [8–11]. This suggests that *UBAP1* mutations are a relatively frequent cause of HSP, and that *UBAP1* warrants inclusion on current HSP gene testing panels.

### Overlap with Other Inherited Disorders

**Fig. 1:**
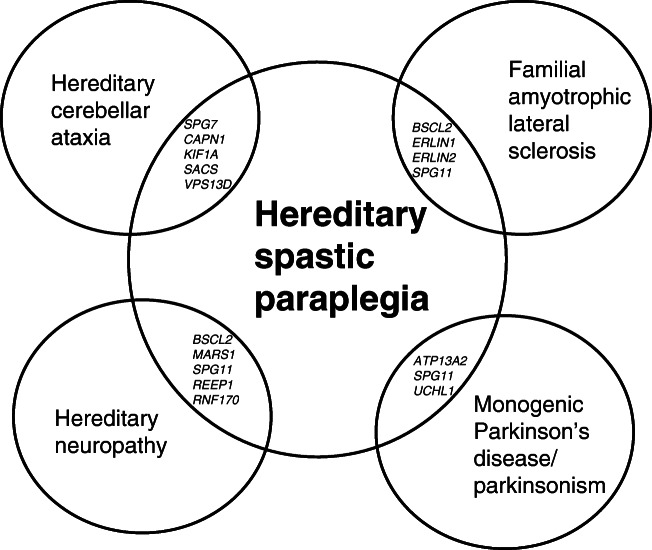
Examples of overlapping genes and shared phenotypes with hereditary spastic paraplegia

There is a large overlap between HSP and other disorders such as inherited forms of hereditary ataxia, peripheral neuropathy, amyotrophic lateral sclerosis (ALS) and Parkinson’s disease. Twenty-eight of 81 genetic forms of HSP are assigned alternative phenotypes on OMIM (Table 1) and this presents further diagnostic complexity (Fig. 1). Genetic testing is often performed with gene panels that are tailored to a specific disease category, and therefore an accurate clinical classification becomes a critical step able to significantly influence the diagnostic yield.

#### Overlap with the Hereditary Cerebellar Ataxias

Inherited ataxias commonly overlap with HSP [19], with a typical example being SPG7 [20, 21]. *SPG7* mutations result in mitochondrial dysfunction [22] and may present with ataxia evolving to spastic ataxia phenotypes, as well as other features such as ophthalmoplegia and ptosis. SPG7 accounted for 2.3% of cerebellar ataxia cases in an Italian population [23]. Similarly, mutations in *CAPN1* cause HSP with or without ataxia [24–27]. It has been suggested that ataxia and spasticity should not be considered separate phenotypes, but rather as existing on a ‘continuous ataxia-spasticity disease spectrum’ [19]. *KIF1A* mutations can cause both HSP and ataxia phenotypes (discussed below) [28•]. Mutations in *SACS* cause autosomal recessive spastic ataxia of Charlevoix-Saguenay (ARSACS), a disorder characterised by the triad of cerebellar ataxia, peripheral neuropathy, and spasticity; however not all features of the triad may be present and there is a phenotypic overlap with the AR HSP with a thin corpus callosum (AR-HSP-TCC) [29]. *VPS13D* mutations cause a recessive ataxia-spasticity spectrum movement disorder [30] but have also been reported to cause a pure or complicated form of HSP (Table 1) [31]. Additionally, HSP-like phenotypes can also be caused by expansions in triplet-repeat ataxia loci [32] and thus, may not be detected on a sequencing panel.

#### Overlap with the Inherited Neuropathies

Many forms of HSP overlap with the inherited neuropathies. Notable examples include mutations in *BSCL2*, which cause Silver syndrome, a complicated form of HSP in which affected individuals present with early-onset hand muscle wasting and leg spasticity [33]. *BSCL2* mutations can also cause a range of phenotypes with lower motor neurone involvement including multifocal motor neuropathy with conduction block, Charcot-Marie-Tooth neuropathy type 2 and distal hereditary motor neuropathy type V [33, 34]. *SPG11* mutations are a major cause of AR-HSP-TCC [35], but may also cause AR Charcot-Marie Tooth disease [36]. Mutations in *MARS1* cause AR HSP complicated by cognitive impairment and nephrotic syndrome [37], as well as AD Charcot-Marie-Tooth Disease type 2 U [38]. Mutations in *REEP1*, the cause of SPG31, have been shown to cause distal hereditary motor neuropathy type V (Table 1). Recessive *RNF170* mutations have recently been confirmed as a cause of HSP [14, 39]**, but a heterozygous mutation in *RNF170* (p.Arg199Cys) was found to cause autosomal dominant late-onset progressive sensory ganglionopathy as a cerebellar ataxia, neuropathy, and vestibular areflexia syndrome (CANVAS) mimic [40].

#### Overlap with Hereditary Amyotrophic Lateral Sclerosis

There are many shared genes between HSP and ALS. For example, mutations in *ERLIN1* have been implicated in SPG62, but may also be the cause of a slowly progressive early-onset ALS [41]. *ERLIN2* mutations, causing SPG18, can evolve into rapidly progressive ALS [42] or cause juvenile primary lateral sclerosis [43]. Notably, Erlin1 and erlin2 are highly homologous endoplasmic reticulum membrane proteins that assemble into a ring-shaped complex [44]. Other examples of HSP genes implicated as causing ALS phenotypes include *SPG11* [45] and *BSCL2* [34].

#### Overlap with Monogenic Parkinson Disease

SPG11 has been linked with parkinsonism or dystonia-parkinsonism. This is highlighted by a recent study which showed that disruption of presynaptic dopaminergic pathways was a widespread phenomenon in individuals with *SPG11* mutations, even without clinical manifestations of parkinsonism [46]. Of note, patients were unresponsive to levodopa, a finding which may relate to post-synaptic damage [46].

Recently, *ATP13A2* mutations have been described as a cause of HSP complicated by cognitive impairment, cerebellar ataxia, and axonal motor and sensory polyneuropathy (SPG78) [47]. Mutations in this gene were first reported as a cause of an AR form of early-onset parkinsonism with pyramidal degeneration and dementia known as Kufor-Rakeb syndrome [48].

There has been a suggestion of a link between *UCHL1* and Parkinson’s disease [49], although this association has not been confirmed. Mutations in *UCHL1* have subsequently been implicated in an early-onset neurodegenerative syndrome, which may be considered HSP complicated by optic atrophy, cerebellar ataxia, seizures, myotonia, fasciculations, dorsal column signs, facial dysmorphism, myopathic facies, microcephaly and fasciculations [50, 51].

### HSP Mimics: Other Mendelian Causes and Management Implications

HSP may be due to mutations in many other genes outside of the SPG loci, typically causing complicated phenotypes. For example, pathogenic variants in *OPA3* can cause an optic atrophy plus syndrome, characterised by optic atrophy and lower limb spasticity [52]. Mutations in *PEX16* have been shown to cause HSP complicated by cerebellar ataxia and dystonia [53, 54]. *TUBB4A* mutations have been initially described as a cause of whispering dysphonia (DYT4 dystonia) [55], but have subsequently been reported as a cause of HSP [56].

Several of the HSP mimics may be neurometabolic disorders with whose timely diagnosis has relevant implications for therapeutic strategies and management [57]. These disorders may have distinctive clinical features and biochemical findings (Table 2). Important examples include mutations in *ABCD1*, the gene associated with adrenoleukodystrophy and adrenomyeloneuropathy, which can cause spastic paraplegia in males and carrier females [24•]. Dopa-responsive dystonia may be misdiagnosed as HSP and is typically responsive to levodopa therapy [58]. Recently, combined homocysteinaemia with methylmalonic aciduria due to pathogenic recessive variants in the *MMACHC* gene has been highlighted as a treatable cause of HSP [59]. Testing urine methylmalonic acid and serum homocysteine levels and sequencing the *MMACHC* gene is critical when this rare condition is suspected [59]. Severe 5,10-methylenetetrahydrofolate reductase deficiency has also been reported as a cause of a complicated HSP phenotype, responsive to treatment with betaine and vitamins [60]. Additionally, cerebrotendinous xanthomatosis may mimic HSP and is treatable with chenodeoxycholic acid [61].

**Table 2:** Examples of ‘treatable’ inherited mimics in HSP

Disorder	Genetic basis	Mode of inheritance	Additional clinical features	Biochemical findings	Treatment	References
Adrenoleukodystrophy, MIM 300100; adult adrenomyeloneuropathy, MIM 300100	*ABCD1*	XLR	Sphincter disturbances, sexual dysfunction, adrenocortical dysfunction	Elevated very long chain fatty acids	Corticosteroid replacement therapy for adrenal insufficiency	Kim et al. [24•], Raymond et al. [62]
Argininemia, MIM 207800	*ARG1*	AR	Dystonia, dementia, peripheral neuropathy, epilepsy	Newborn screening, elevation of plasma arginine concentration	Measures to reduce ammonia, such as protein-restricted diet, branched-chained amino acids supplement and sodium benzoate.	Tsang et al. [63]
Biotinidase deficiency, MIM 253260	*BTD*	AR	Seizures, hypotonia, limb weakness, ataxia, developmental delay, visual impairment, hearing loss, cutaneous abnormalities	Newborn screening or deficient biotinidase enzyme activity in serum/plasma	Treatment with biotin	Wolf [64], Wolf [65]
Primary coenzyme Q10 deficiency 8, MIM	*COQ7*	AR	Primary coenzyme Q10 (CoQ10) deficiency is usually associated fatal neonatal encephalopathy with hypotonia, multiple-system atrophy-like phenotype, dystonia, spasticity, seizures, intellectual disability, sensorineural hearing loss, steroid-resistant nephrotic syndrome, hypertrophic cardiomyopathy	Reduced levels of CoQ10 in skeletal muscle or reduced activities of complex I+III and II+III of the mitochondrial respiratory chain on frozen muscle homogenates	2,4-Dihydroxybenzoate bypass treatment, high-dose oral CoQ_10_ supplementation	Wang et al. [66], Salviati et al. [67]
Cerebrotendinous xanthomatosis, MIM 213700	*CYP27A1*	AR	Cerebellar signs, intellectual impairment, seizures, peripheral neuropathy, cataract, tendon xanthomas	Elevated levels of cholestanol and bile alcohols in serum and urine	Chenodeoxycholic acid	Nicholls et al. [61], Verrips et al. [68]
DOPA-responsive dystonia, MIM 128230	*GCH1*	AD, AR	Foot dystonia, later development of parkinsonism, diurnal variation in symptoms, dramatic and sustained response to levodopa	Reduced concentrations of total biopterin and total neopterin in the cerebrospinal fluid	Levodopa/decarboxylase inhibitor	Fan et al. [58]
Methylmalonic aciduria and homocystinuria cblC type MIM 277400	*MMACHC*	AR	Cognitive impairment (5/8), spastic dysuria (3/8), personality change and depression (3/8), ataxia (2/8), seizures (2/8), limb numbness (2/8) and developmental delay (2/8). When patients were diagnosed, the mean serum homocysteine level, the methylmalonic acid level in urine, the serum propionylcarnitine (C3) level and the ratios of C3-to-acetylcarnitine (C2) and free carnitine (C0) were all dramatically elevated. Cranial MRIs showed nothing remarkable except mild brain atrophy.	Elevated urine methylmalonic acid and serum homocysteine levels	Intramuscular cobalamin, oral betaine and folate	Wei et al. [59]
Homocystinuria due to MTHFR deficiency, MIM 236250	*MTHFR*	AR	Polyneuropathy, behavioural abnormalities, cognitive impairment, psychosis, seizures, leukoencephalopathy	Severe hyperhomocysteinemia associated with the characteristic amino acid profile	Betaine and vitamins	Lossos et al. [60]
Phenylketonuria, MIM 261600	*PAH*	AR	Cognitive impairment	Serum phenylalanine concentrations	Classic phenylketonuria diet/protein restricted diet	Kasim et al. [69]
Dystonia 9, MIM 601042; GLUT1 deficiency syndrome 1, MIM 606777; GLUT1 deficiency syndrome 2, MIM 612126; Stomatin-deficient cryohydrocytosis with neurologic defects, MIM 608885	*SLC2A1*	AD	Seizures, delayed neurologic development, acquired microcephaly, intermittent ataxia, paroxysmal exercise-induced dyskinesia, choreo-athetosis, alternating hemiplegia	Cerebrospinal fluid analysis for hypoglycorrhachia	Ketogenic diet	Verrotti et al. [70]

*AD*, autosomal dominant; *AR*, autosomal recessive; *XLR*, X-linked recessive

### HSP Mimics: Overlap with Disorders Without Clear Mendelian Inheritance

Several disorders that do not have a readily recognisable monogenic cause may be difficult to differentiate from HSP, such as primary lateral sclerosis (PLS). PLS is a degenerative, mainly sporadic neuronopathy with primarily upper motor neurone features [71]. PLS frequently presents with spastic paraplegia, affects older, predominantly male patients and invariably progresses to involve cervical and bulbar regions [71]. However, the disease often remains as an isolated spastic paraplegia for many years and bulbar symptoms can appear after 10 years in up to 20% of patients [71]. Consequently, in the absence of family history, PLS and HSP may be clinically indistinguishable for longer than a decade [71]. However, cortical excitability studies may be used to differentiate these two conditions in a clinical setting [72], and genetic testing for HSP genes may also help [73].

It may also be challenging to differentiate between HSP and cerebral palsy. HSP may be distinguishable from spastic diplegic cerebral palsy by the absence of perinatal risk factors for brain injury and normal brain imaging, or specific findings indicative of an HSP syndrome, such as thinning of the corpus callosum [74]. Genetic testing may also be helpful, for example, a patient with childhood onset, non-progressive, spastic diplegia with no previous family history of HSP was long considered as affected by cerebral palsy, until his son also developed the same phenotype: genetic testing in these patients disclosed a heterozygous pathogenic variant in *ATL1* (SPG3A) which had arisen de novo in the affected parent [75].

There may also be diagnostic uncertainty in differentiating HSP from multiple sclerosis. A personal observation is that patients may be referred to the neurogenetics clinic with HSP, only to find evidence of demyelinating lesions consistent with MS on upon repeating brain or spinal cord MRI. Conversely, mutations in HSP genes may be identified in individuals formerly diagnosed with MS. For example, SPG2 has been shown to mimic MS [76], and rare variants in genes including *KIF5A* and *REEP1* were identified in patients with primary progressive MS [77].

### When Should a Complex Disorder Be Diagnosed as HSP?

It may be difficult to decide when to categorise a disorder as HSP when the phenotype is complex. A chief consideration should be whether lower extremity weakness and spasticity are the *predominant* clinical manifestations [78]. For example, *ATP13A2* mutations are known to cause Kufor-Rakeb syndrome [48], neuronal ceroid lipofuscinosis [79] and neurodegeneration with brain iron accumulation (NBIA) [80]. More recently, *ATP13A2* mutations have been described as a cause of HSP complicated by cognitive impairment, cerebellar ataxia, and axonal motor and sensory polyneuropathy (SPG78) [47]. However, there is debate over whether an HSP predominant phenotype is a clinical outlier and if a new HSP locus was warranted [81]. Similarly, hypomorphic mutations in *POLR3A* were reported as a cause of HSP and ataxia [82], however, other authors considered that this condition should be defined as a ‘POLR3-related disorder’ instead [83].

### HSP Genes with Different Modes of Inheritance

Variants in some HSP genes may be inherited with different modes of transmission, adding further complexity to the interpretation of genetic findings. As an example, biallelic mutations in *KIF1A* cause spastic paraplegia, distal wasting, peripheral neuropathy and mild cerebellar signs (AR SPG30) [84]. *KIF1A* mutations can also cause hereditary sensory and autonomic neuropathy type 2 with AR inheritance (Table 1). However, de novo dominant *KIF1A* mutations may result in a phenotypic spectrum overlapping with AR SPG30 including mental retardation, speech delay, epilepsy, optic nerve atrophy, thinning of the corpus callosum, periventricular white matter lesion and microcephaly [85–88]. A recent study showed that heterozygous mutations in *KIF1A* may result in two distinct phenotypes, a pure to complex HSP phenotype and a congenital or early-onset ataxia phenotype [28•]. Additionally, mutations in *REEP2* have been identified in families with both AD and AR inheritance [37, 89]. A mutation in *REEP2* has been found to cause AD HSP with a pure, early-onset phenotype [89], while the AR form is characterised by early-onset HSP with delayed motor milestones and normal cognition [37]. Similarly, *ATL1* mutations are usually associated with dominant HSP (SPG3A), but recessive mutations in *ATL1* have been shown to cause both pure and complex forms of HSP [90, 91].

### Individuals with Concurrent Independent Genetic Diagnoses

Individuals presenting with HSP may have concurrent independent genetic diagnoses, further complicating genetic testing. As an example, a recent study showed two possible genetic diagnoses in a non-consanguineous family with 3 affected siblings: two brothers with intellectual impairment and spastic paraplegia, and a sister with behavioural disturbance and pes cavus. All affected siblings carried a maternally inherited interstitial 15q duplication and a paternally inherited *REEP1* variant [92•]. In this case, it was thought that the 15q duplication was causing intellectual impairment and behavioural abnormalities, with supportive evidence from methylation and functional studies. On the other hand, the dominant HSP phenotype was attributed to the *REEP1* variant. This in keeping with a large study of 7374 consecutive unrelated patients referred to a clinical diagnostic laboratory for WES, which demonstrated multiple molecular diagnoses in 4.9% of cases in whom WES was informative [93]. The results of these studies suggest that perhaps we too often claim a ‘phenotypic expansion’ to explain a phenotype that is different or more complicated than previously reported for a given gene, while in some of these cases the reason would be a ‘double hit’ and not a phenotypic expansion.

### Pseudodominant Inheritance and Intronic Variants

In a recent study, a patient with spastic paraplegia and ataxia was investigated with WES, revealing a novel missense variant in *SPG7* (c.2195T>C; p.Leu732Pro) [94•]. To seek a second variant, WGS was performed, revealing an unreported, deep intronic variant (c.286 + 853A>G), shown to activate a cryptic splice site [94•]. The deep intronic variant would not have been identified with WES alone, highlighting the usefulness of WGS to increase diagnostic yield [94•]. Furthermore, it sheds light on the apparent dominant pattern of inheritance of SPG7 [95], which may be due to the mutation on the other allele being missed [94•]. Another report highlights the importance of an intronic variant in *POLR3A*, a gene previously associated with hypomyelinating leukodystrophy type 7 (Table 1), as a frequent cause of HSP and cerebellar ataxia [82]. Compound heterozygous mutations in *POLR3A* were found in approximately 3.1% of index cases of HSP and cerebellar ataxia, with over 80% carrying the same intronic mutation (c.1909+22G>A) which activates a cryptic splice site [82]. This suggests that non-coding DNA variants may account for a substantial number of unsolved cases of HSP.

## Strategies for a Genetic Diagnosis

There are several options for reaching a genetic diagnosis in individuals with HSP and it can be challenging for the clinician to decide upon which approach to adopt. Different strategies include targeted sequencing gene panels, whole-exome sequencing (WES), or whole-genome sequencing (WGS) (Table 3). Targeted sequencing gene panels are commonly used but will overlook a diagnosis if the mutation is in a gene that is outside the panel. Furthermore, gene panels also are not reliable in detecting copy number variants (CNVs), structural variants (SVs) and intronic variants. WES can be a useful approach but again may not be reliable for CNVs, SVs, and will fail to detect deep intronic variants. WGS may be the most complete approach [24•, 54, 96], with uniformity of coverage that allows for the accurate detection of CNVs, SVs [97, 98], in addition to the detection of non-coding variants. However, this approach is limited by the expense and difficulty processing, storing, and interpreting the large amounts of genomic data. Both WES and WGS allow for testing of many genes and so may not be restricted to single panel, e.g. patients can be tested for both ‘ataxia’ and ‘HSP’ genes in a single test [24•]. The WES and WGS data can be used in several ways. For example, a panel of relevant HSP genes may be analysed, such as those listed in Table 1. A larger, less specific panel of genes can also be interrogated, such as the TruSight One ‘clinical exome’—a panel of 4813 genes that have been associated with human disease [99]. WES or WGS family studies may provide valuable additional information regarding segregation of genetic variants with the disease phenotype. For example, parent-child trios of healthy parents and an affected child may facilitate the detection of homozygous, compound heterozygous or de novo variants.

**Table 3:** Comparison of different approaches for the genetic diagnosis of hereditary spastic paraplegia

Technique	Pros	Cons
Targeted sequencing panels	- Less expensive* - Reduce incidental findings	- Gene list may be restrictive, missing unexpected findings or mutations in genes implicated in overlapping phenotypes - Inadequate coverage of CNVs, SVs; MLPA may be required - Inadequate coverage of deep non-coding variants
Whole-exome sequencing	- Gene panel not restrictive - Less expensive compared to whole-genome sequencing*	- Inadequate coverage of CNVs, SVs; MLPA may be required s- Inadequate coverage of deep non-coding variants - Challenge of incidental findings
Whole-genome sequencing	- Gene panel not restrictive - Detection of CNVs, SVs (e.g. deletions in *SPAST*) - Detection of non-coding variants (see example of deep intronic variants reported in *SPG7*)	- Expensive* - Challenge of processing, storing and analysing large amounts of data - Challenge of incidental findings

*Note that to our knowledge, a cost-effectiveness study for genetic testing in hereditary spastic paraplegia comparing the different approaches has not yet been performed.

It is critical to remember that CNVs (e.g. exonic deletions in *SPAST* [100]) are important to consider and may require a separate test (e.g. multiplex ligation probe amplification or MLPA), unless using a method that provides reliable detection such as WGS. Furthermore, testing for repeat expansion disorders will often require a separate test such as a fluorescent repeat-primed PCR assay. However, a recent study suggests that long repeat expansions may be detectable from PCR-free WGS data using a software tool called ExpansionHunter [101]. Furthermore, a homoplasmic m.9176 T>C mutation in the mitochondrial *ATP6* gene has been found to cause HSP. WES may allow for the detection of mitochondrial point mutations using ‘off-target reads’, providing additional diagnoses [102]. WGS provides exceptionally high coverage of the mitochondrial genome, allowing for accurate detection of mitochondrial point mutations even at low levels of heteroplasmy [103]. Hypothesis-free methods such as WES and WGS may also detect multiple concurrent genetic defects, as described above [92•].

## Benefits of a Genetic Diagnosis in HSP

There are numerous benefits of a genetic diagnosis in HSP which may prompt the decision to undertake genetic testing. As an example, it may provide for prognostic information and facilitate genetic counselling and family planning. It may also allow for a prenatal diagnosis/preimplantation genetic diagnosis.

A genetic diagnosis rarely leads to findings with direct management implications (as discussed earlier, see Table 2). However, it may hold future value in that it could be used to enrol patients in clinical trials that target the disease mechanism. A targeted, disease-modifying treatment appears most likely for two forms of HSP—SPG4 and SPG5.

Microtubule-targeting drugs hold great promise for HSP due to *SPAST* mutations (SPG4). Supporting this concept, vinblastine has been shown to ameliorate the disease phenotype in a *Drosophila* model of SPG4 [104]. Additionally, microtubule-targeting drugs have been shown to rescue axonal swellings in cortical neurons in a mouse model of SPG4 [105]. In human patient-derived olfactory neurosphere-derived cells, *SPAST* mutations result in decreased levels of acetylated α-tubulin, a marker of stabilised microtubules, as well as reduced speed of peroxisome trafficking [106]. Tubulin binding drugs such as taxol, vinblastine, epothilone D and noscapine may increase acetylated alpha tubulin and thereby restore axonal transport, directly targeting the mechanism involved in SPG4 [106, 107].

Several genes associated with HSP phenotypes disturb lipid metabolic pathways as a potential therapeutic target, including *CYP7B1*, *EPT1*, *PCYT2*, *DDHD1*, *DDHD2*, *PNPLA6*, *B4GALNT1*, *CYP2U1*, *FA2H*, *GBA2*, *PLA2G6*, *ATP13A2*, *BSCL2*, *C19orf12*, *ERLIN2*, *SPART*, *SPAST*, *SPG11*, *SPG15*, *ATL1* and *REEP1* [108]. SPG5 is a recessive cause of HSP due to mutations in the *CYP7B1* gene encoding a distinct microsomal oxysterol-7α-hydroxylase. This enzyme is involved in the degradation of cholesterol into primary bile acids. CYP7B1 deficiency results in accumulation of neurotoxic oxysterols, with elevation of 25-hydroxycholesterol (25-OHC) and 27-hydroxycholesterol (27-OHC) in the plasma and a much higher increase of 27-OHC in the CSF [109, 110]. Two recent studies have explored the use of drugs to lower cholesterol biomarkers in HSP. A study by Marelli and colleagues used atorvastatin, chenodeoxycholic acid and resveratrol in 21 patients with SPG5A and assessed 25-OHC and 27-OHC as diagnostic biomarkers [111••]. Treatment with atorvastatin decreased plasma 27-OHC but did not change the 27-OHC to total cholesterol ratio or 25-OHC levels. Marelli and colleagues also identified an abnormal bile acids profile in patients with SPG5, with a reduction in total serum bile acids and a decrease of ursodeoxycholic and lithocholic acids in comparison to deoxycholic acid. Treatment with chenodeoxycholic acid restored the bile acid profile. The authors concluded that atorvastatin and chenodeoxycholic acid may be worth considering for the treatment of SPG5A. A randomised placebo control trial by Schols and colleagues found that atorvastatin treatment reduced 27-OHC and 25-OHC in the serum, although 27-OHC was not significantly reduced in the cerebrospinal fluid [109••]. It is important to note that both these trials have demonstrated a reduction in cholesterol/bile acid biomarkers, but without benefit in terms of clinical, imaging, or electrophysiological outcome measures.

A more recent study explored the use of intravenous formulated mouse and human CYP7B1 mRNA in mice lacking the endogenous *Cyp7b1* gene mutated in SPG5A. Results indicated that the treatment was safe and demonstrated a reduction in neurotoxic oxysterols in the liver, serum and to some degree in the brain, suggesting that this may be a valid strategy for the treatment of this condition [112].

## Conclusion

The genetic diagnosis of HSP is complex and can represent a major challenge for clinicians. The complexity arises in part because of the high degree of genetic heterogeneity, with over 80 different genetic forms, and a growing number of genes being identified. Furthermore, there is a high level of phenotypic complexity, with HSP clinically and genetically overlapping with a variety of neurological phenotypes, including inherited forms of cerebellar ataxia, ALS, Parkinson’s disease, and peripheral neuropathy. There are many conditions that mimic HSP that the clinician should be alert for, and it may be particularly important to detect the rare HSP mimics that have management implications.

An understanding of the genetic and phenotypic complexity underlying HSP is essential to guide genetic testing strategies. Gene panels are commonly used, but the gene panel itself needs to be comprehensive to encompass the large number of genes involved. Panels must be regularly curated given that the rapid rate of gene discovery as they can quickly become obsolete. Furthermore, gene panels are typically based on a specific phenotypic category, and a genetic diagnosis may be missed if the responsible mutation is in a gene outside of that disease category. Thus, directed testing approaches such as gene panels may miss unanticipated findings [54].

Hypothesis-free approaches such as clinical WES, WES and WGS somewhat overcome the potential problems of gene panels by allowing for the potential interrogation of many relevant genes. However, clinical WES and WES may miss certain mutation types such as CNVs, SVs and repeat expansions, potentially detectable with WGS. In fact, WGS may be the most comprehensive method for coverage and detection of mutation types but is unfortunately limited by cost.

Next-generation sequencing has greatly improved our ability to detect a genetic diagnosis in HSP. Yet, large studies have shown that the diagnostic rate for HSP is still only about 45–50% of cases [113, 114]. The genetic diagnosis of HSP still represents a great challenge for clinicians, and there are no clear guidelines available about which approach to choose. However, it will become increasingly important to identify a genetic diagnosis in a rapid and accurate manner for enrolment in clinical trials and as targeted treatments become available.
